# A Polyamine-Based Dinitro-Naphthalimide Conjugate as Substrates for Polyamine Transporters Preferentially Accumulates in Cancer Cells and Minimizes Side Effects *in vitro* and *in vivo*

**DOI:** 10.3389/fchem.2020.00166

**Published:** 2020-04-09

**Authors:** Jing Ma, Yingguang Li, Linrong Li, Kexin Yue, Hanfang Liu, Jiajia Wang, Zhuoqing Xi, Man Shi, Sihan Zhao, Qi Ma, Sitong Liu, Shudi Guo, Jianing Liu, Lili Hou, Chaojie Wang, Peng George Wang, Zhiyong Tian, Songqiang Xie

**Affiliations:** ^1^School of Pharmacy, Institute for Innovative Drug Design and Evaluation, Henan University, Kaifeng, China; ^2^Joint National Laboratory for Antibody Drug Engineering, School of Basic Medicine Science, Henan University, Kaifeng, China; ^3^Henan University of Science and Technology Second Affiliated Hospital, Luoyang, China; ^4^School of Medicine, Henan University Minsheng College, Kaifeng, China; ^5^The Key Laboratory of Natural Medicine and Immuno-Engineering, Henan University, Kaifeng, China; ^6^Southern University of Science and Technology, School of Medicine, Shenzhen, China

**Keywords:** dinitro-naphthalimide conjugate, polyamine, polyamine transporter, cancer, minimized side-effects

## Abstract

Naphthalimides, such as amonafide and mitonafide in clinical trials, have been developed as antitumor agents for orthotopic tumor. However, the serious side effects in cancer patients limit their applications. Herein, a new class of polyamine-based naphthalimide conjugates **5a-5c**, **7a-7b**, and **11a-11b** with and without the alkylation of the distant nitrogen in the polyamine chain were synthesized and the mechanism was determined. Compared with amonafide, dinitro-naphthalimide conjugate **5c** with a 4,3-cyclopropyl motif preferentially accumulates in cancer cells and minimizes side effects *in vitro* and *in vivo*. More importantly, **5c** at the dosage of as low as 3 mg/kg (57.97%) displays better antitumor effects than the positive control amonafide (53.27%) at 5 mg/kg *in vivo*. And a remarkably elevated antitumor activity and a reduced toxicity are also observed for **5c** at 5 mg/kg (65.90%). The upregulated p53 and the apoptotic cells (73.50%) indicate that the mechanism of **5c** to induce apoptosis may result from its enhanced DNA damage. Further investigation indicates that in addition to target DNA, **5c** can modulate the polyamine homeostasis by upregulating polyamine oxidase (PAO) in a different way from that of amonafide. And also by targeting PTs overexpressed in most of cancer cells, **5c** downregulates the contents of Put, Spd, and Spm, which are in favor of suppressing fast-growing tumor cells. Our study implies a promising strategy for naphthalimide conjugates to treat hepatic carcinoma with notable activities and reduced toxicities at a low dosage.

## Introduction

As the sixth most prevalent malignancy, hepatocellular carcinoma (HCC) is a kind of cancer that is found too late and has a high mortality worldwide (Forner et al., 2012; Dou et al., 2016). Approximately 90% of cancer patients with HCC cannot survive for more than 5 years even after the treatment by anticancer drugs (Chen et al., 2016; Siegel et al., 2016). In spite of advances in the rational and combinatorial technologies for cancer therapy, current anticancer agents can only cure parts of recurrence and metastasis cells after tumor excision at high doses, which can lead to severe side effects. (Li et al., 2016) Therefore, it is urgent to develop novel drugs with enhanced activities and reduced toxicities at a relatively low dosage.

**Figure 1:**
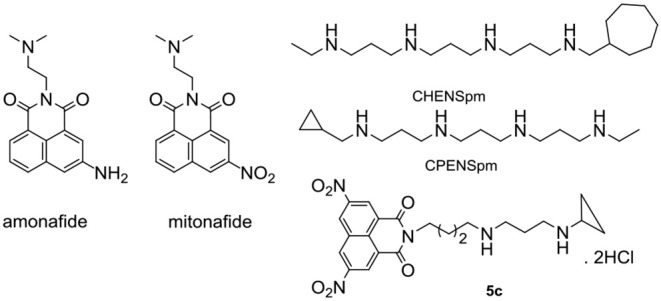
Chemical structures of amonafide, mitoafide, polyamine analogs CHENSpm, and CPENSpm, and target compound **5c**.

Recently, except for photophysical characters, a surge in the activities of naphthalimides has also been developed as versatile functional compounds for promising antitumor activities (Banerjee et al., 2013; Chen et al., 2013; Seifert et al., 2016; Mateusz and Krzysztof, 2018; Peddaboodi et al., 2018; Yulin et al., 2019). Amonafide and mitonafide in Figure 1 as the representative naphthalimides have entered clinical trials (Stone et al., 2015). One of the major reasons why amonafide and mitonafide cannot be used widely is because they can have serious side effects in cancer patients. Thus, the design of novel naphthalimide therapeutic agents with reduced toxicity represents an area that is in need of urgent attention.

Resurgence in the interest of natural polyamines as an anticancer strategy results from advances in our understanding of polyamine metabolism and their alterations in cancer (Casero and Marton, 2007; Casero et al., 2018; Phanstiel, 2018). Natural products with polyamine moiety have been found to be a promising strategy to enhance targeting properties and deduce the toxicities (Muth et al., 2013, 2014; Skruber et al., 2017). Polyamine analogs CHENSpm and CPENSpm in Figure 1 have entered in clinical trials. Our group (Wang et al., 2012; Li et al., 2016, 2018; Dai et al., 2017a; Ma et al., 2017a,b; Ma et al., 2018; Liu et al., 2019) focused on polyamine-based naphthalimide conjugates to achieve enhanced pharmacological effects. And polyamine-based naphthalimide conjugates are also used to treat HCC as mitochondria or lysosome targeting antitumor and antimetastatic agents. So far, the main modification of naphthalimides is focused on amino and nitro groups, which play an important role in the antitumor activity. Unfortunately, polyamine-based di-amino and di-nitro naphthalimide conjugates are virtually unexplored. Moreover, in the structure of CHENSpm and CPENSpm, the alkylation of the distant nitrogen in the polyamine chain plays an important role in increasing the activity in cancer cells and decreasing the toxicity in normal cells. Our group and others find that natural products with homospermidine moiety without the alkylation of the distant nitrogen in the polyamine chain showed good activities (Casero and Marton, 2007; Wang et al., 2012; Muth et al., 2013, 2014; Li et al., 2016, 2018; Dai et al., 2017a; Ma et al., 2017a,b, 2018; Skruber et al., 2017; Casero et al., 2018; Phanstiel, 2018; Liu et al., 2019). However, naphthalimide conjugates with the alkylation of the distant nitrogen in the polyamine chain are rarely reported.

**Scheme 1:**
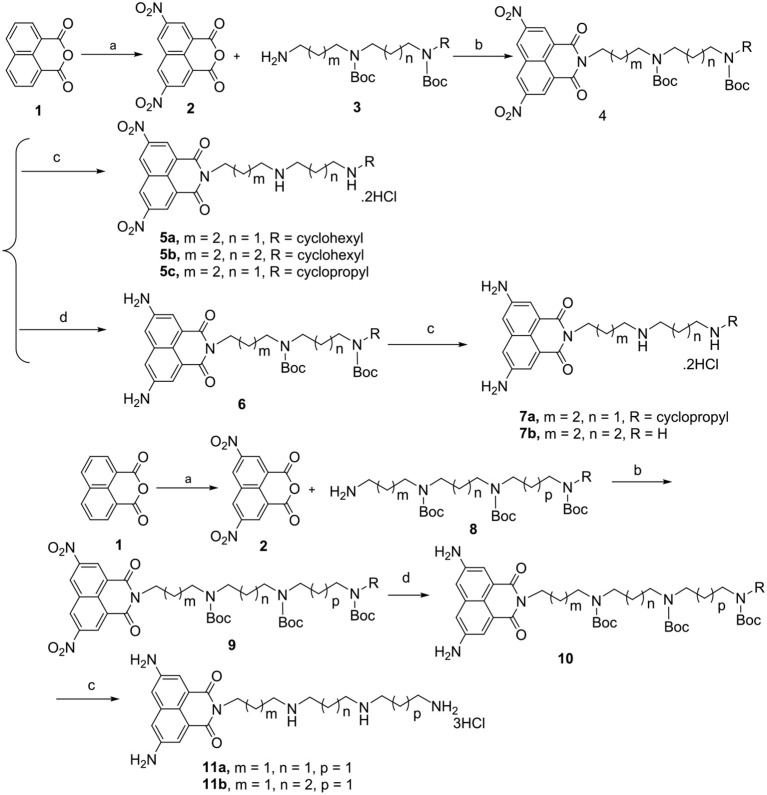
Chemical structures and synthetic route of polyamine-based naphthalimide conjugates **5a-5c**, **7a-7b**, and **11a-11b** in yield of 25–30%. Reagents and conditions: (a) HNO_3_, 50°C, 3 h; (b) K_2_CO_3_, CH_3_CN, 85°C, 5 h; (c) EtOH, 4 M HCl, rt, overnight; (d) Pd/C, H_2_, MeOH, rt, 2 h.

Based on the key role of amino and nitro group in mediating antitumor efficacy of naphthalimide conjugates, we firstly designed and synthesized a new class of polyamine-based di-nitro and di-amino naphthalimide conjugates **5a-5c**, **7a-7b**, and **11a-11b** with and without the alkylation of the distant nitrogen in the polyamine chain. For the first time, we summarized the structure-activity relationship (SAR) of **5a-5c**, **7a-7b**, and **11a-11b** with and without the classical unsymmetrically-substituted polyamine analogs CHENSpm and CPENSpm (Casero and Marton, 2007; Casero et al., 2018; Phanstiel, 2018) (Figure 1 and Scheme 1). Chains with different lengths, such as 4,4 and 4,3-cyclohexyl or 4,3-cyclopropyl substituted diamine, or 3,3,3 and 3,4,3 substituted triamine motif are also selected for the construction of **5a-5c**, **7a-7b**, and **11a-11b** to investigate different lengths on antitumor activity. Amonafide was selected as the positive control. We established feasible routes to **5a-5c**, **7a-7b**, and **11a-11b** in Scheme 1.

## Experimental Section

### General Procedure for Obtaining Title Compounds, Using 5a as Example

The known intermediate **2** was prepared by a conventional method using concentrated nitric acid in glacial acetic acid (Gryshchenko et al., 2015; Soriano et al., 2016). A solution containing **3** (1 mmol) in CH_3_CN (5 mL) at 0°C was added to a solution of **2** (1 mmol) and K_2_CO_3_ in CH_3_CN (10 mL). Then the mixture was heated to 85°C for 5 h. After monitoring by Thin-Layer Chromatography (TLC), the reaction mixture was cooled to room temperature and concentrated under vacuum to give an oily residue. After extraction and purification by column chromatography with dichloromethane/methanol (100:1–100:3, v/v) as the elution solvent, **4** was obtained in a yield of 60%. At 0°C we added four molar HCl (2 mL), and then **4** in CH_3_CH_2_OH (2 mL) was stirred at room temperature overnight until a great amount of precipitate was generated. The filtered cake was washed by anhydrous CH_3_CH_2_OH, dried to give the target compound **5a** as a hydrochloride salt in a yield of 56%. ^1^H NMR (300 MHz, DMSO-d6) δ = 9.76 (s, 2H), 9.09 (s, 2H), 2.97 (m, 2H), 2.50 (m, 7H), 1.64 (m, 16H). ^13^C NMR (75 MHz, D_2_O) δ = 163.75, 147.26, 131.96, 130.76, 127.12, 124.38, 57.50, 47.29, 44.43, 41.26, 40.13, 28.83, 24.45, 24.12, 23.87, 23.10, 22.85. ESI-MS (positive ion mode): m/z [M]^+^: calcd: 497.07; obsd: 498.07. Calcd for C_25_H_33_Cl_2_N_5_O_6_: C 52.64%, H 5.83%, N 12.28%. Found: C 52.58%, H 5.73%, N 12.19%.

### Dinitro-Naphthalimide Conjugate 5b

**5b** was obtained according to the procedure of **5a** except for replacing **3** with 4,4 -cyclohexyl substituted diamine as shown in Scheme S1 in a yield of 65%. ^1^H NMR (300 MHz, D_2_O) δ = 9.31 (s, 2H), 9.09 (s, 2H), 4.03 (m, 2H), 3.01 (m, 7H), 2.04–0.94 (m, 19H). ^13^C NMR (75 MHz, D_2_O) δ = 163.25, 147.18, 131.79, 130.60, 126.95, 124.17, 57.20, 47.11, 46.78, 43.58, 40.19, 28.97, 24.45, 24.13, 23.86, 23.10, 22.99, 22.83. ESI-MS (positive ion mode): m/z [M]^+^: calcd: 511.06; obsd: 512.06. Calcd for C_26_H_35_Cl_2_N_5_O_6_: C 53.43%, H 6.04%, N 11.98%. Found: C 53.36%, H 5.98%, N 12.19%.

### Dinitro-Naphthalimide Conjugate 5c

**5c** was obtained according to the procedure of **5a** except for replacing **3** with 4,3-cyclopropyl substituted diamine as shown in Scheme S1 in a yield of 68%. ^1^H NMR (300 MHz, D_2_O) δ = 9.39 (s, 2H), 9.17 (s, 2H), 4.12 (s, 2H), 3.39–3.06 (m, 6H), 2.75 (d, *J* = 4.2, 1H), 2.30–2.01 (m, 2H), 1.81 (m, 4H), 0.90 (dd, *J* = 18.3, 7.2, 4H). ^13^C NMR (75 MHz, D_2_O) δ = 163.28, 147.16, 131.83, 130.72, 127.02, 124.09, 47.32, 44.96, 44.49, 40.22, 30.09, 24.15, 23.15, 22.47. ESI-MS (positive ion mode): m/z [M]^+^: calcd: 455.00; obsd: 456.00. Calcd for C_22_H_27_Cl_2_N_5_O_6_: C 50.01%, H 5.15%, N 13.25%. Found: C 49.98%, H 5.09%, N 13.19%.

### Diamino-Naphthalimide Conjugate 7a

Intermediate **4** was obtained according to the procedure as described in **5a**. Then Pd/C was added to the solution of **4** dissolved in MeOH (10 ml) and stirred in hydrogen at room temperature for 2 h to obtain **5** in a yield of 85%. Then, 4 M HCl was added according to our previous report (Wang et al., 2012; Li et al., 2016, 2018; Dai et al., 2017a; Ma et al., 2017a,b; Ma et al., 2018; Liu et al., 2019) to give the pure target compound **7a** in a yield of 68%. ^1^H NMR (300 MHz, D_2_O) δ = 8.20–7.77 (s, 2H), δ = 720–6.77 (s, 2H), 4.00 (d, *J* = 6.6, 2H), 3.18–2.84 (m, 6H), 2.61 (s, 1H), 1.90 (t, *J* = 29.9, 2H), 1.68 (m, 4H), 0.77 (t, *J* = 12.5, 4H). ^13^C NMR (75 MHz, D_2_O) δ = 164.33, 133.95, 132.56, 124.45, 123.69, 123.25, 47.34, 47.09, 45.37, 37.56, 30.04, 24.27, 22.88, 22.61, 2.96. ESI-MS (positive ion mode): m/z [M]^+^: calcd: 395.04; obsd: 396.04 Calcd for C_32_H_69_Cl_5_N_6_O_5_Pt: C 38.81%, H 7.02%, N 8.49%. Found: C 38.90%, H 7.09%, N 8.40%. Calcd for C_22_H_31_Cl_2_N_5_O_2_: C 56.41%, H 6.67%, N 14.95%. Found: C 56.39%, H 6.59%, N 14.85%.

### Diamino-Naphthalimide Conjugate 7b

**7b** was obtained according to the procedure of **7a** except for replacing **3** with 4,4-unsubstituted diamine as shown in Scheme S1 in a yield of 69%. ^1^H NMR (300 MHz, D_2_O) δ = 7.86–7.43 (m, 2H), 3.80 (d, *J* = 6.7, 2H), 3.24–2.72 (m, 6H), 1.64 (m, 8H). ^13^C NMR (75 MHz, D_2_O) δ = 164.14, 136.89, 132.73, 122.48, 122.27, 121.17, 47.23, 46.89, 39.83, 38.84, 24.21, 23.96, 23.21, 22.78. ESI-MS (positive ion mode): m/z [M]^+^: calcd: 369.00; obsd: 370.00. Calcd for C_20_H_29_Cl_2_N_5_O_2_: C 54.30%, H 6.61%, N 15.83%. Found: C 53.98%, H 6.58%, N 15.79%.

### Diamino-Naphthalimide Conjugate 11a

**11a** was obtained according to the procedure of **7a** except for replacing **3** with **8** as shown in Scheme S2 in a yield of 62%. ^1^H NMR (300 MHz, D_2_O) δ = 7.79 (d, *J* = 35.7, 2H), 3.91 (d, *J* = 6.1, 2H), 3.01 (m, 10H), 2.21–1.85 (m, 6H). ^13^C NMR (75 MHz, D_2_O) δ = 166.53, 137.02, 134.91, 126.45, 126.28, 125.50, 125.28, 48.09, 47.25, 47.17, 47.11, 40.06, 39.08, 26.77, 26.26, 25.23. ESI-MS (positive ion mode): m/z [M]^+^: calcd: 398.05; obsd: 399.05 Calcd for C_21_H_33_Cl_3_N_6_O_2_: C 49.66%, H 6.55%, N 16.55%. Found: C 49.56%, H 6.45%, N 16.45%.

### Diamino-Naphthalimide Conjugate 11b

**11b** was obtained according to the procedure of **11a** except for replacing **3** with **8** as shown in Scheme S2 in a yield of 62%. ^1^H NMR (300 MHz, D_2_O) δ = 8.04–7.55 (m, 2H), 4.20–2.47 (m, 12H), 2.26–1.49 (m, 8H). ^13^C NMR (75 MHz, D_2_O) δ = 164.35, 135.19, 132.56, 123.61, 122.78, 122.72, 122.61, 47.03, 45.45, 44.56, 37.52, 36.58, 24.25, 23.75, 22.98, 22.80. ESI-MS (positive ion mode): m/z [M]^+^: calcd: 412.06; obsd: 413.06. Calcd for C_22_H_35_Cl_3_N_6_O_2_: C 50.63%, H 6.76%, N 16.10%. Found: C 50.58%, H 6.68%, N 15.98%.

### *In vitro* Cellular Cytotoxicity Assays

We incubated hepatic carcinoma (Snu-368 and Snu-739), breast carcinoma (MDA-MB-231 and MCF-7), cisplatin-sensitive lung cancer cells A549, and cisplatin-resistant lung cancer cells A549cisR in 96-well plates in a 5% CO_2_ atmosphere at 37°C for 24 h (5,000 cells/well). After adding drugs in freshly prepared culture medium (100 μl) and incubating for another 48 h, we added 20 μl MTT (5 mg/ml) and incubated for another 3 h. At last, after the medium was removed, 150 μl DMSO was added. By using a Bio-Rad 680 microplate reader, the absorbance was measured at 570 nm and the IC_50_ values were calculated using the GraphPad Prism software based on three parallel experiments.

### Inhibitory Effects of 5c on Snu-368 and Snu-739 Cells With and Without Spd (50 μM) After 24-h Treatment

Inhibitory effects of **5c** on Snu-368 and Snu-739 cells (5000 cells/well) were conducted similar to the MTT assay except that the Spd-containing RPMI medium was used for serial dilution of the compound-containing concentrated solutions.

### Determination of the Contents of PAO (Polyamine Oxidase)

We measured the total PAO level using a PAO assay kit according to our previous report (Liu et al., 2019) (Hepeng Biotechnology, Cat. HEPENGBIO156).

### Western Blot Assay

We performed western blot analysis to determine the contents of p53 after treatment by **5c** for 5, 10, and 15 μM for 24 h. After washing three times with PBS, we harvested and centrifuged the cells, which were lysed with a RIPA buffer (Beyotime, China). The total contents of protein were determined by a BCA assay kit (Beyotime, China). At 100°C for 10 min, the total lysates were denatured in a 5 × SDS-loading buffer. Equal amounts of total proteins were separated by 12% SDS-PAGE. Dried skimmed milk (5%) was used to block the separated protein in Tris Buffered Saline Tween (TBST) at room temperature for 1 h. And then the corresponding primary antibodies and the appropriate HRP-conjugated secondary antibody were used to incubate with the separated protein. By ECL plus reagents (Beyotime, Jiangsu, China), we detected the expression of p53.

### Polyamines Contents Assay

According to our previous report (Wang et al., 2012; Li et al., 2016, 2018; Dai et al., 2017a; Ma et al., 2017a,b, 2018; Liu et al., 2019), the contents of Put, Spd, and Spm were detected by a G1321A fluorescence detector in both Snu-368 and Snu-739 cells. Cells were harvested and dansyl chloride was used as the derivation reagent. After converting to the corresponding dansyl derivatives, Put, Spd, and Spm were separated by using High Performance Liquid Chromatogra (HPLC) (Agilent 1260, Agilent Technologies, USA) with a C18 chromatographic column (25 × 4.6 mm, 5 μm). As the internal standard substance, 1,6-diaminohexane was used. The excitation and emission wavelengths were 340 and 515 nm, separately. The mobile phase was methanol–water from 65:35 to 100:0 within 30 min.

### Annexin V-FITC/Propidium Iodide Staining

Annexin V-FITC/PI staining was used to detect the apoptosis of Snu-739 cells by FCM (flow cytometry). Firstly, we plated the cells in six-well plates (1 × 10^5^ cells/well). **5c** 10 μM and amonafide 10 μM were added and incubated for 24 h. And then we harvested the cells and washed three times with PBS. Then the protocol was stained according to our previous report (Wang et al., 2012; Li et al., 2016, 2018; Dai et al., 2017a; Ma et al., 2017a,b; Ma et al., 2018; Liu et al., 2019) and was performed by FCM (BD Biosciences, San Jose, CA, USA).

### *In vivo* Antitumor Assays

Healthy BALB/c mice (Cat. SCXK 2016-0006, Beijing, China) aged 5 weeks were used for the determination of anticancer activity. We obtained BALB/c mice from the Laboratory Animal Center, Academy of Military Medical Science. They were raised in compliance with the Guide for the Care and Use of Laboratory Animals. The weight of healthy BALB/c mice is 18–22 g.

We firstly injected HCC cells with 1 × 10^6^ cells per mouse to determine the anticancer activity. After a week, the tumors increased to 80–120 mm^3^. The mice were divided into four groups for **5c** (3 mg/kg), **5c** (5 mg/kg), the positive control amonafide (5 mg/kg), or the negative control physiological saline. We injected **5c** or amonafide *via* the tail vein every day for a total of seven treatments. Body weight was determined every day. And then the mice were sacrificed after 7 days. The tumor tissues were weighed and the inhibition rate [((average tumor weight of negative control group – average tumor weight of the drug treated or positive control group)/average tumor weight of control group) × 100] was calculated. The organ index [(organ weight/body weight) × 100%] was also counted including heart, liver, kidney, lung, and spleen at the last day. Both **5c** and amonafide were dissolved in glucose injection. And they were used immediately after preparation.

The ethical committee approved the projects *in vivo* with the number HUSOM-2016-316, and we performed all animal procedures following the protocol approved by the Institutional Animal Care and Use Committee at Henan University.

## Results

### Synthesis and Characterization of Polyamine-Based Naphthalimide Conjugates 5a-5c, 7a-7b, and 11a-11b

Feasible routes to **5a**-**5c**, **7a**-**7b**, and **11**a-**11b** (Scheme 1) were established and given in detail in the Supporting Information. The linkers **3** and **8** (Schemes S1,S2) were first prepared by the conventional reaction of 2-(4-bromobutyl)isoindoline-1,3-dione **a** or 2-(3-bromopropyl)iso-indoline-1,3-dione **i** as the starting materials to obtain the hydrophobic polyamine chain varying connecting formats from 4,4 and 4,3-cyclohexyl or cyclopropyl substituted diamine, to 3,3,3 and 3,4,3 substituted triamine motif in a yield of 65%. We also established the detail on the feasible routes of the linkers **3** and **8** in the Supporting Information. We prepared known intermediate **2** by a conventional method using concentrated nitric acid in glacial acetic acid (Gryshchenko et al., 2015; Soriano et al., 2016). Generated intermediate **2** reacted with diverse protected polyamines **3** and **8** to give compounds **4** and **9**. **6** and **10** were obtained by the reduction reaction with Pd/C in the presence of hydrogen in MeOH. **4**, **6**, and **10** were deprotected with 4 M HCl to provide target compounds **5a-5c**, **7a-7b**, and **11a-11b** as hydrochloride salts in a yield of 25–30%.

By ^1^H, ^13^C NMR spectroscopy, ESI-MS (Figures S2–S24) and CHN elemental analysis, all new compounds were characterized. We confirmed the purity of platinum complexes **4-7** ≥ 95% by HPLC (Tables S1,S2).

### *In vitro* Cytotoxicity Effects

*In vitro* assays were firstly conducted to evaluate the inhibitory effect of polyamine-based naphthalimide conjugates **5a-5c**, **7a-7b**, and **11a-11b** on six cancer cell lines, namely, Snu-368 and Sun-739 (hepatoma cell line), MCF-7 (breast carcinoma), MDA-MB-231 (triple negative breast cancer), cisplatin-sensitive lung cancer A549, and resistant A549cisR cells by using traditional MTT tests. Two classic antitumor agents in clinic trials, amonafide and cisplatin, were chosen as the reference drugs (Table 1). The preliminary structure–activity relationship (SAR) can be obtained from the *in vitro* biological results.

**Table 1:** IC_50_ values (μM) of polyamine-based naphthalimide conjugates **5a-5c**, **7a-7b**, and **11a-11b**, the positive control amonafide, and cisplatin by MTT assays^[d]^.

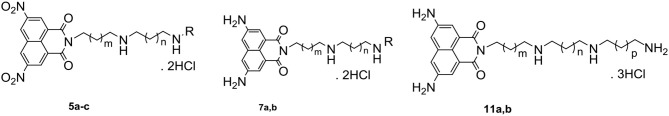											
					**IC**_**50**_ **(μM)**						
	***R*****=**	***m*****=**	***n*****=**	***p*****=**	**Snu-368**	**Snu-739**	**MDA-MB-231**	**MCF-7**	**A549**	**A549cisR**	**RF**^[a]^
**5a**	Cyclohexyl	2	1	/	2.94 ± 0.06	2.16 ± 0.10	1.19 ± 0.12	2.3 ± 0.18	2.17 ± 0.20	3.62 ± 0.15	1.67
**5b**	Cyclohexyl	2	2	/	2.72 ± 0.25	3.89 ± 0.35	1.07 ± 0.10	1.25 ± 0.10	1.75 ± 0.15	2.27 ± 0.25	1.30
**5c**	Cyclopropyl	2	1	/	1.09 ± 0.10	0.76 ± 0.05	1.33 ± 0.12	1.35 ± 0.15	1.92 ± 0.23	0.83 ± 0.08	0.43
**7a**	Cyclopropyl	2	1	/	>30	25.77 ± 2.90	26.91 ± 2.65	8.62 ± 0.85	13.98 ± 1.39	17.38 ± 1.68	/
**7b**	H	2	2	/	>30	>30	>30	>30	>30	17.30 ± 1.78	/
**11a**	/	1	1	1	>50	9.69 ± 0.98	25.96 ± 2.56	14.81 ± 1.45	28.3 ± 2.85	16.33 ± 1.69	/
**11b**	/	1	2	1	>30	23.17 ± 2.36	>30	25.91 ± 2.56	>30	15.81 ± 1.58	/
Amonafide					13.98 ± 1.78	12.98 ± 1.56	14.89 ± 1.75	5.89 ± 0.56	6.89 ± 0.68	10.98 ± 1.02	1.59
FI^[b]^					12.83	17.08	11.20	4.36	3.59	13.23	3.70
Cisplatin					16.37 ± 1.26	10.02 ± 1.23	15.98 ± 1.59	9.60 ± 0.60	11.00 ± 0.15	40.36 ± 4.36	3.67
FI^[c]^					15.02	13.18	12.02	7.11	5.73	48.63	8.74

[a] *The RF (resistance factor) is defined as the IC_50_ value in A549cisR cells/IC_50_ value in A549 cells*.

[b] *FI (fold increase) is defined as IC_50_(amonafide)/IC_50_(**5c**)*.

[c] *FI (fold increase) is defined as IC_50_(cisplatin)/IC_50_(**5c**)*.

[d] *An average of three measurements. ND = not determined*.

Compounds **5a-5c**, **7a-7b**, and **11a-11b**, with a different methylene linker, exhibited different potency in the tested cancer cells, indicating that the linker in these polyamine conjugates plays an important role in their antitumor activities.

Dinitro-naphthalimide conjugates **5a**-**5c** displayed more potent antitumor activities than compounds **7a-7b** and **11a-11b** with diamino moieties. For dinitro-based scaffolds (**5a**-**5c**), **5c** with a 4,3-cyclopropyl substituted diamine motif was the most active toward all six of the tested cancer cells, which was more potent than its 4,3-cyclohexyl (**5a**) and 4,4-cyclohexyl (**5b**) counterparts. Furthermore, **5c** (FI 3.59–48.63) was significantly more active than amonafide and cisplatin with some potencies in the nanomolar range. For the first time, we found that polyamine-based dinitro-naphthalimide conjugates **5a**-**5c** (RF 0.43–1.67) can overcome cisplatin resistance efficiently, the RF (resistance factor) values of which are 2–9-folds lower than that of cisplatin (RF = 3.67). Among these compounds, **5c** displayed the highest cytotoxicity. And then **5c** was focused for the following tests.

### *In vitro* Cytotoxicity Effects on Cancer Cells and the Matched Normal Cells

The selectivity for cancer cells over normal healthy cells is important for ideal anticancer agents, thereby mitigating undesired toxic side effects associated with chemotherapy. The selectivity of polyamine-based dinitro-naphthalimide conjugates **5a**-**5c** was evaluated by Snu-368 and Snu-739 (hepatic carcinoma), and the matched normal cells HL-7702 (normal liver cell). Moreover, dinitro-naphthalimide conjugates **5a**-**5c** (SI 1.94–7.47) show lower cytotoxicity in normal cells.

**Figure 2:**
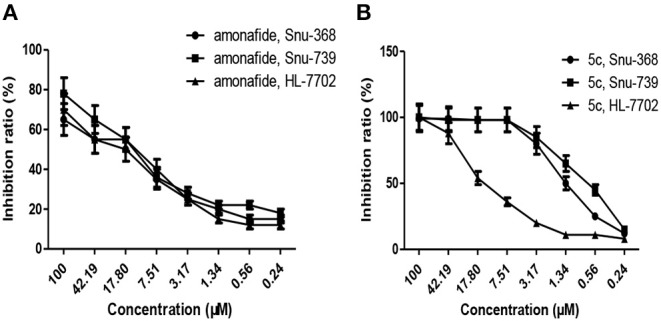
Inhibition ratio of amonafide **(A)** and **5c (B)** on cancer cells (Snu-368 and Snu-739) and the matched normal cells (HL-7702) with 10 μM of tested complexes.

Notably, as presented in Table 2, the IC_50_ values of **5c** (SI 5.21–7.47) were significantly lower in the cancer cells compared to the matched normal cells, SI of which is 8–33-folds higher than that of amonafide (SI 0.83–0.89) and cisplatin (SI 0.53–0.86). A similar tendency was observed for the inhibition ratio of **5c**, amonafide, and cisplatin with 10 μM of tested complexes (Figure 2).

**Table 2:** Inhibitory effect (IC_50_ in μM) of dinitro-naphthalimide conjugates **5a-5c**, amonafide, and cisplatin on cancer cells Snu-368 and Snu-739 (hepatic carcinoma), and the matched normal cells HL-7702 (normal liver cell).

	**Snu-368**	**Snu-739**	**HL-7702**	**SI^**[a]**^**	**SI^**[b]**^**
**5a**	2.94 ± 0.06	2.16 ± 0.10	6.30 ± 0.23	2.14	2.92
**5b**	2.72 ± 0.25	3.89 ± 0.35	7.56 ± 0.43	2.78	1.94
**5c**	1.09 ± 0.10	0.76 ± 0.05	5.68 ± 0.83	5.21	7.47
Amonafide	13.98 ± 1.78	12.98 ± 1.56	11.56 ± 1.36	0.83	0.89
Cisplatin	16.37 ± 1.26	10.02 ± 1.23	8.60 ± 0.20	0.53	0.86

[a] *SI(selectivity index) is defined as IC_50_ in HL-7702/IC_50_ in Snu-368*.

[b] *SI(selectivity index) is defined as IC_50_ in HL-7702/IC_50_ in Snu-739*.

For the first time, we found that the polyamine-based dinitro-naphthalimide conjugate **5c** displayed the highest cytotoxicity to cancer cells and reduced toxicity to normal cells *in vitro*. Therefore, we focus on **5c** for the following tests. And also the results of antitumor activity *in vitro* prompted us to further test the *in vivo* antitumor activities.

### Tumor Growth Inhibition *in vivo*

**Figure 3:**
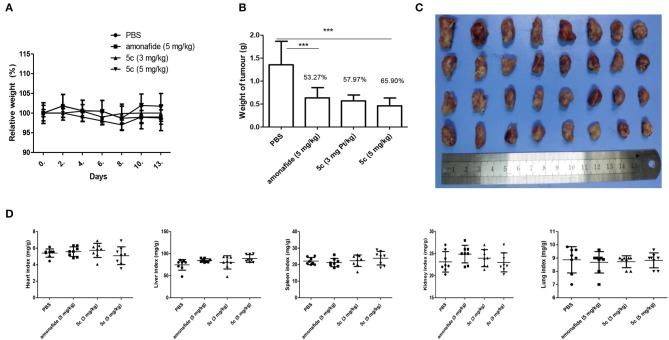
*In vivo* antitumor activity of **5c** and amonafide in HCC tumors. Eight mice each received the vehicle, amonafide (5 mg/kg), **5c** (3 mg/kg), or **5c** (5 mg/kg). **(A)** Relative weight of the mice for PBS, amonafide, and **5c** group. **(B)** Tumor weight for PBS, amonafide, and **5c** group at the end of the experiment. **(C)** Images of the tumors for PBS, amonafide, and **5c** group at the end of the experiment. First horizontal line, control group; second line, amonafide group (5 mg/kg); third line, **5c** group (3 mg/kg); fourth line, **5c** group (5 mg/kg). **(D)** Organ weight indexes [(organ weight/body weight) × 100%] including heart, liver, kidney, lung, and spleen were calculated for PBS, amonafide, and **5c** group at the end of the experiment. ***P < 0.001.

The lowest IC_50_ of **5c** (0.76 μM) in Snu-739 cells (Table 1) indicates its better therapeutic effects in HCC. The highest FI levels in Snu-739 also indicate a significant therapy for HCC. Therefore, HCC animal models were used to test the *in vivo* antitumor activities. Mice bearing HCC xenografts were treated with **5c** (3 mg/kg), **5c** (5 mg/kg), the positive control amonafide (5 mg/kg), and normal saline (negative control) once every day for 7 days by tail vein (Figure 3).

The tumor suppression of **5c** at the dosage of 3 mg/kg (57.97%) and 5 mg/kg (65.90%) was better than that of amonafide (53.27%, 5 mg/kg). On day 7, the average tumor volume (900 mm^3^) for the control group was much higher than that of the **5c** group (480 mm^3^), indicating enhanced antitumor activity *in vivo*. Meanwhile, the variations of organ weight indexes implied that **5c** showed no obvious pathological changes in the experiments of toxicological profile *in vivo* (Figure 3D), which was consistent with the effects of **5c** *in vitro*.

### Induction of Apoptosis

**Figure 4:**
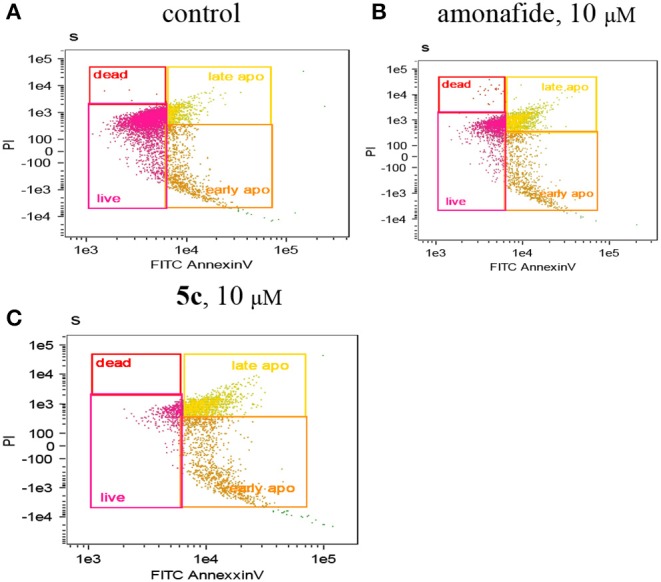
The apoptotic percentage in Snu-739 cells was detected by FCM with **(C)** or without **(A)** 5c (10 μM) or amonafide **(B)** (10 μM) for 24 h.

Next, FCM experiments using Annexin V/PI double staining were conducted to further determine if apoptosis was induced by DNA damage (Figure 4). We can see that both the early apoptotic cells (35.00%) and late apoptotic cells (38.50%) of **5c** (10 μM) were higher than that of amonafide (10 μM). This indicated that the enhanced toxicity of dinitro-naphthalimide conjugate **5c** may result from the DNA damage.

**Figure 5:**
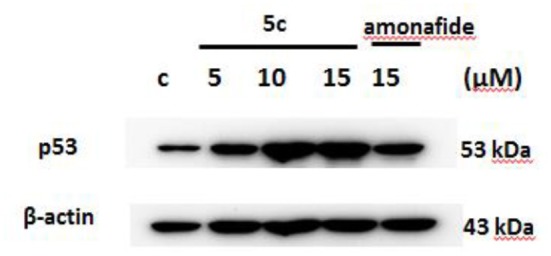
The expression of p53 in Snu-739 cells after treatment with **5c** and amonafide.

The p53 protein expression in Snu-739 cells was tested to further determine the DNA damage in Figure 5. The upregulated p53 after treatment with **5c** indicates that the mechanism of **5c** to induce apoptosis may result from its enhanced DNA damage.

### Polyamine Transporters (PTs) Were Partially Involved in the Cellular Entrance of 5c

**Figure 6:**
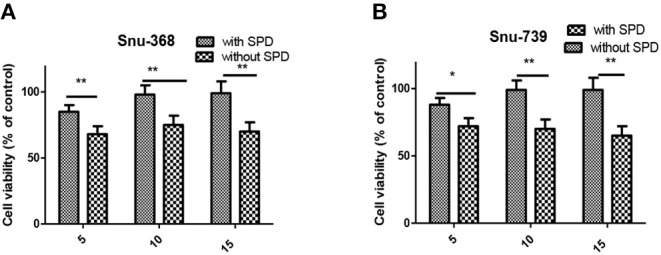
Cell viability of Snu-368 **(A)** and Snu-739 **(B)** cells after treatment with 5c was determined with and without Spd (50 μM). ***P < 0.001.

PTs, overexpressed in most of cancer cells, is vital to polyamine-conjugate. Next, cell viability of Snu-368 and Snu-739 cells was calculated with and without the PTs inhibitor Spd (Figure 6). We observed that in the presence of 20 μM Spd cell viability increased 20–30% in both Snu-368 and Snu-739 cells. Hypothesis was confirmed that in the cellular entrance of **5c** PTs were at least partially involved.

### 5c Affects Polyamine Metabolism and Function by Upregulating Polyamine Oxidase (PAO)

**Figure 7:**
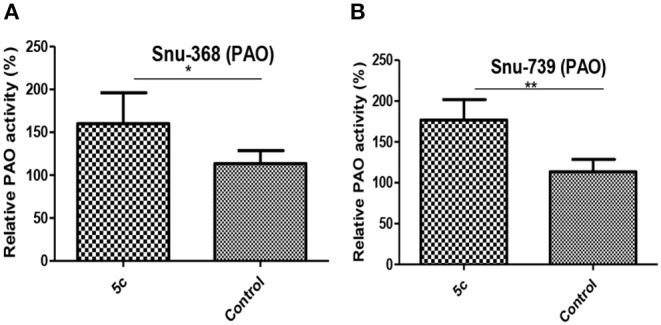
Effect of **5c** on PAO expression in Snu-368 **(A)** and Snu-739 **(B)** cells with 10 μM after 24 h treatment. **P < 0.05; **P < 0.01.

PAO is a critical catabolism enzyme in polyamine metabolism. The upregulation of PAO can influence tumor high polyamine microenvironment to induce a significant accumulation of ROS, which can also promote apoptosis (Wang et al., 2013; Chen et al., 2017; Dai et al., 2017b). We found that the relative PAO activity in Snu-368 and Snu-739 cells upon **5c** treatment is 1.5–2-folds higher than the control group (Figure 7).

The elevated PAO can upregulate oxidizing substances such as ROS and downregulate reducing substances such as GSH, which can lead to cisplatin resistance. Herein, the upregulated PAO is also believed to be among the major reasons for **5c** to overcome cisplatin resistance.

**Figure 8:**
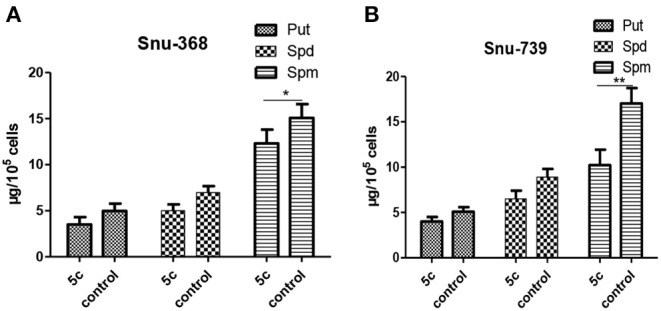
Put, Spd, and Spm concentrations in Snu-368 **(A)** and Snu-739 cells **(B)** after 24 h treatment with or without 5c was determined. ^*^P < 0.05; ^**^P < 0.01.

And the contents of Put, Spd, and Spm can be decreased by the upregulated PAO to promote polyamine metabolism and suppress fast-growing tumor cells. Next, the contents of Spm, Spd, and Put in Snu-368 and Snu-739 cells were measured (Figure 8). Compared with the control group, **5c** downregulated the contents of Put, Spd, and Spm in Snu-368 and Snu-739 cells.

## Conclusions

**Figure 9:**
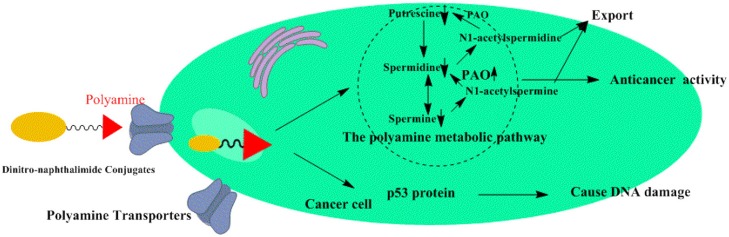
Proposed mechanism of action for dinitro-naphthalimide conjugates **5c**.

Taken together, our findings provide the first example of polyamine-based dinitro-naphthalimide conjugate **5c** as substrates for PTs preferentially accumulate in cancer cells and minimize side effects *in vitro* and *in vivo*. By targeting polyamine catabolic enzyme PAO by PTs, **5c** downregulates Put, Spd, and Spm to regulate tumor high polyamine microenvironment (Figure 9). And upregulating the p53 protein **5c** causes significant DNA damage to induce apoptosis. The discovery of the potential role of dinitro-naphthalimide conjugate implies a promising strategy for naphthalimide conjugates with targeting properties to treat hepatic carcinoma with notable activities and reduced toxicities at a low dosage.

## Data Availability Statement

The raw data supporting the conclusions of this article will be made available by the authors, without undue reservation, to any qualified researcher.

## Ethics Statement

The animal study was reviewed and approved by Henan University.

## Author Contributions

JM, CW, PW, JW, ZT, and SX contribute to the conception, design, analysis, and writing of the study against the collection of data and other routine work. HL, YL, LL, and KY contribute to the synthesis of compounds in this paper. LH, ZX, MS, SZ, QM, SL, SG, and JL contribute to the mechanism research and animal experiments *in vitro* and *in vivo*. LH contributed to the analysis, interpretation of data for this work.

### Conflict of Interest

The authors declare that the research was conducted in the absence of any commercial or financial relationships that could be construed as a potential conflict of interest.
