# Supplementary material for: A Polyamine-Based Dinitro-Naphthalimide Conjugate as Substrates for Polyamine Transporters Preferentially Accumulates in Cancer Cells and Minimizes Side Effects in vitro and in vivo
Source: Front Chem. 2020 Apr 9;8:166. doi: 10.3389/fchem.2020.00166 (PMC7160362; doi:10.3389/fchem.2020.00166)
Supplement: Supplementary file 1 [file Data_Sheet_1.docx]

Supporting Information

**A Polyamine-based** **Dinitro-****naphthalimide Conjugate as Substrates for Polyamine Transporters Preferentially Accumulates in Cancer Cells and** **Minimizes Side-effects *in vitro* and *in vivo***

Jing Ma,^†#^ Yingguang Li,^†#^ Linrong Li,^†^ Kexin Yue,^†^ Hanfang Liu,^†^ Zhuoqing Xi,^†II^ Man Shi,^†^ Sihan Zhao,^†^ Qi Ma,^†^ Sitong Liu,^†^ Shudi Guo,^†^ Jianing Liu,^I^ Chaojie Wang, ^@^  Peng George Wang, ^IV^ Jiajia Wang, ^III*^ Zhiyong Tian,^†*^ and Songqiang Xie,^&*^

†School of Pharmacy, Institute for Innovative Drug Design and Evaluation, Henan University, N. Jinming Ave, 475004 Kaifeng, China. ^&^School of Pharmacy, Institute of Chemical Biology, Henan University, N. Jinming Ave, 475004 Kaifeng, China. ^III^Henan University Joint National Laboratory for Antibody Drug Engineering; School of Basic Medicine Science, Henan University, Kaifeng, P. R. China. ^@^The Key Laboratory of Natural Medicine and Immuno-Engineering, Henan University, Kaifeng, 475004, China. ^IV^Southern University of Science and Technology, School of Medicine, Shenzhen, China. ^I^School of medicine, Henan University Minsheng College, Kaifeng, 475004, China. ^II^Henan University of Science and Technology Second Affiliated Hospital, Luoyang, 471000, China.

# The authors contribute equally to this work.

**Experimental Procedures**

**Reagents and instruments**

All reagents were used as received without further purification. If necessary, the reactions were conducted in dry solvents and under an argon atmosphere. We purchased cisplatin and oxaliplatin from Shanghai Yurui Chemical Co. Ltd. PAO (Hepeng Biotechnology, Cat. HEPENGBIO156), GSH (Solarbio Biochemical Assay Division, Cat. 20180226) and GSH-Px (Leagene Biotechnology, Cat. 0309A18) Preparation Kit were also purchased. A Genomic DNA Mini Preparation Kit from Beyotime (Cat. 61018010105) was used for cellular drug uptake and DNA platination. All other chemicals were obtained from commercial suppliers, such as Alfa Aesar, Aldrich, J&K, and GL Biochem Ltd. and were of analytical grade. 3-(4,5-dimethylthiazol-2-yl)-2,5-diphenyl tetrazolium bromide (MTT), ascorbic acid (AsA), 5’-dGMP (purity ≥ 98.0%), DMEM and RPMI 1640 medium containing 10% fetal bovine serum were purchased from GL Biochem Ltd. Fetal bovine serum (FBS), 0.25% trypsin/EDTA solution, and penicillin-streptomycin solution were purchased from Invitrogen (Grand Island, NY, USA). The cancer cell lines were cultured in RPMI-1640 medium supplemented with 10% FBS, streptomycin (100 units/mL), and penicillin (100 units/mL) at 37 °C in a humidified atmosphere with 5% CO_2_. These cell lines were purchased from American Type Culture Collection (MD, USA).

A549cisR cells were maintained with 2 μg/mL cisplatin. Phosphate-buffered saline (PBS) contains 137 mM NaCl, 2.7 mM KCl, 10 mM Na_2_HPO_4_ and 2 mM KH_2_PO_4_.

^1^H NMR, and ^13^C NMR spectra were recorded on a Bruker AVANCE AV300 (300 MHz). High-resolution mass spectra (HRMS) were obtained on an IonSpec QFT mass spectrometer with ESI ionization. CHN elemental analysis was carried out on a Vario Micro elemental analyzer. HPLC analyses were performed on a Waters E2695-2998 system equipped with a Venusil MP C18 column (150 × 4.6 mm, 5 μm). HPLC profiles were recorded with a UV detector at 273 nm and a fluorescence detector at an excitation wavelength of 340 nm and an emission wavelength of 515 nm at room temperature. The mobile phase consisted of MeOH and H_2_O at a flow rate of 0.5 mL/min (20 μL injection volume). Reactions involved in the preparation of platinum compounds were conducted in the dark. Platinum levels were examined by inductively coupled plasma mass spectrometry (ICP-MS; PE Elan 6100 DRC or PE Nexion 2000). Genomic DNA was isolated by Genomic DNA Mini Preparation Kit (Beyotime, Cat. 61018010105). DNA level was measured by Nanodrop Spectrophotometer (Thermo Scientific ND-1000) and Pt level was determined by ICP-MS. The Pt level accumulation in cells was also measured by ICP-MS (PE Elan 6100 DRC or PE Nexion 2000) after the overnight digestion of cell pellets with 65% HNO_3_.

**Method for purity determination of target compounds**

The purities of all target compounds were determined by HPLC (Waters E2695-2998 equipped with a Venusil MP C18 column (150 × 4.6 mm, 5 μm). The purity of the platinum complexes (**4**–**7**) were confirmed to be ≥95% by analytical HPLC. The method and purities results of target compounds were as follows (Table S1 and S2).

**Table S1.** Method for determining the purity of target compounds.

| Time (min) | A (water) | B (methanol) |
| --- | --- | --- |
|  | 95% | 5% |
| 20 | 95% | 5% |
| 30 | 50% | 50% |

**Table S2. Purities results of target compounds**

| Compounds | R.T. | Purity(%) |
| --- | --- | --- |
| **5a** | 4.25 | 96.79 |
| **5b** | 4.36 | 98.83 |
| **5c** | 4.46 | 97.69 |
| **7a** | 4.48 | 98.14 |
| **7b** | 4.49 | 97.13 |
| **11a** | 4.46 | 98.23  98.56 |
| **11b** | 4.26 | 96.23 |

**
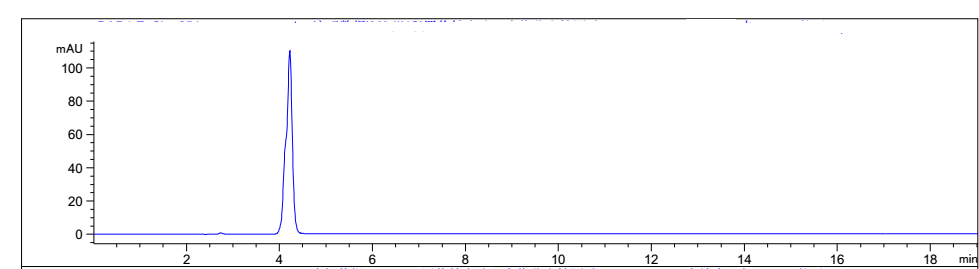
**

**Figure S1.** **5c** in water tested by RP-HPLC at room temperature.

**Data Analysis.**

All the data are presented as the mean ± SD and analyzed using Student’s t test or analysis of variance (ANOVA) followed by q-test.

*p < 0.05, **p < 0.01, and ***p < 0.001 were considered to be statistically significant.

**Results and Discussion**

**Scheme S1**. Synthetic route of polyamine chains **3a-3e**. Reagents and conditions: (i) K_2_CO_3_/CH_3_CN, reflux, 12 h; (ii) (Boc)_2_O, room temperature, overnight; (iii) NH_2_·NH_2_. H_2_O, EtOH, room temperature, overnight.

 **Scheme S2**. Synthetic route of **8a-8b**. Reagents and conditions: (i) K_2_CO_3_/CH_3_CN, reflux, 12 h; (ii) (Boc)_2_O, room temperature, overnight; (iii) NH_2_∙NH_2_. H_2_O, EtOH, room temperature, overnight; (iv) K_2_CO_3_/CH_3_CN, reflux, 12 h; (v) (Boc)_2_O, room temperature, overnight; (vi) NH_2_∙NH_2_. H_2_O, EtOH, room temperature, overnight.


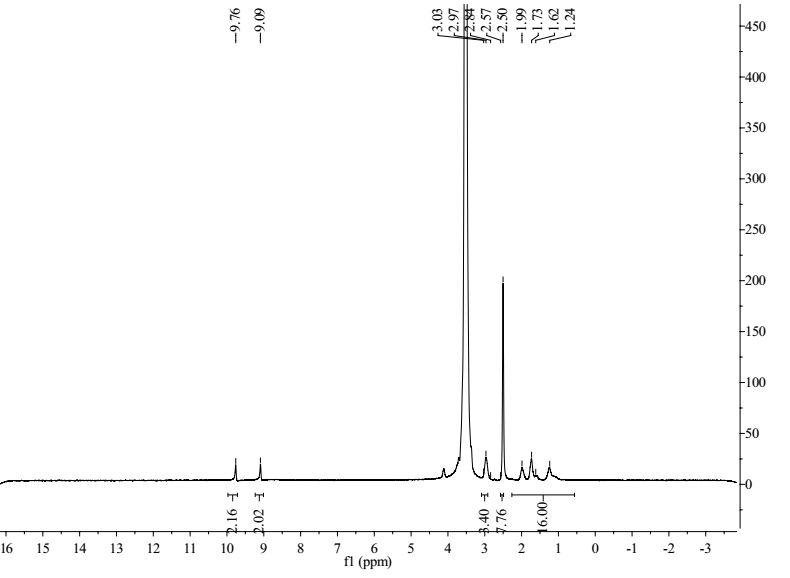


**Figure S2.** ^1^H NMR spectrum of compound **5a**.


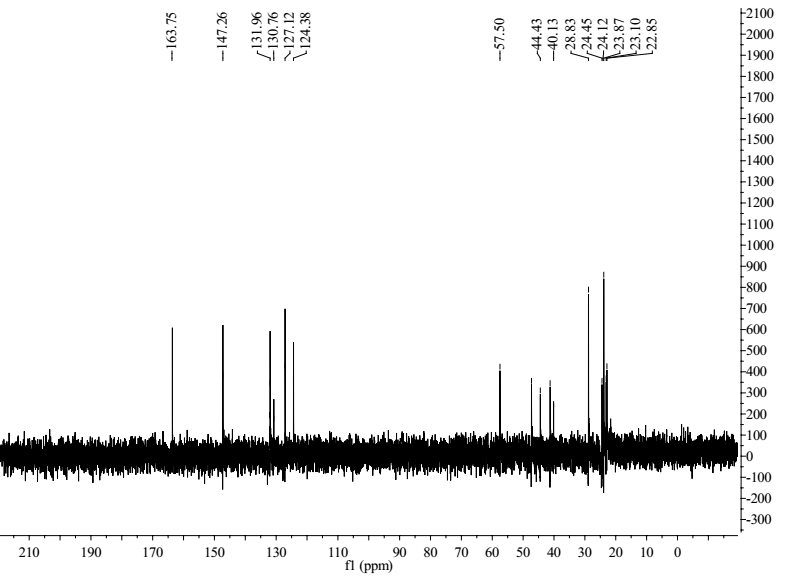


**Figure S3.** ^13^C NMR spectrum of compound **5a**.


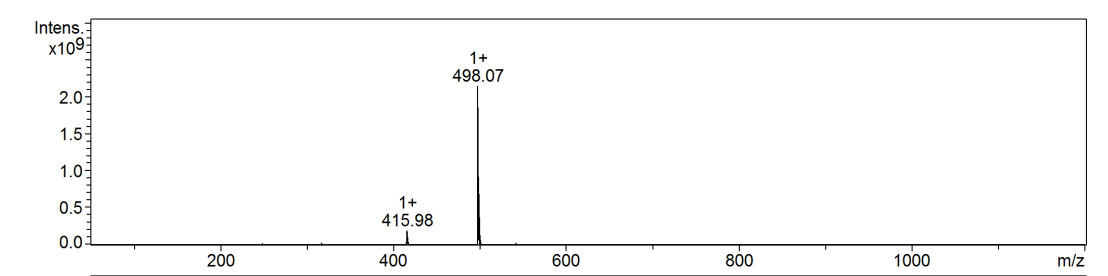


**Figure S4.** ESI mass spectrum of compound **5a**.


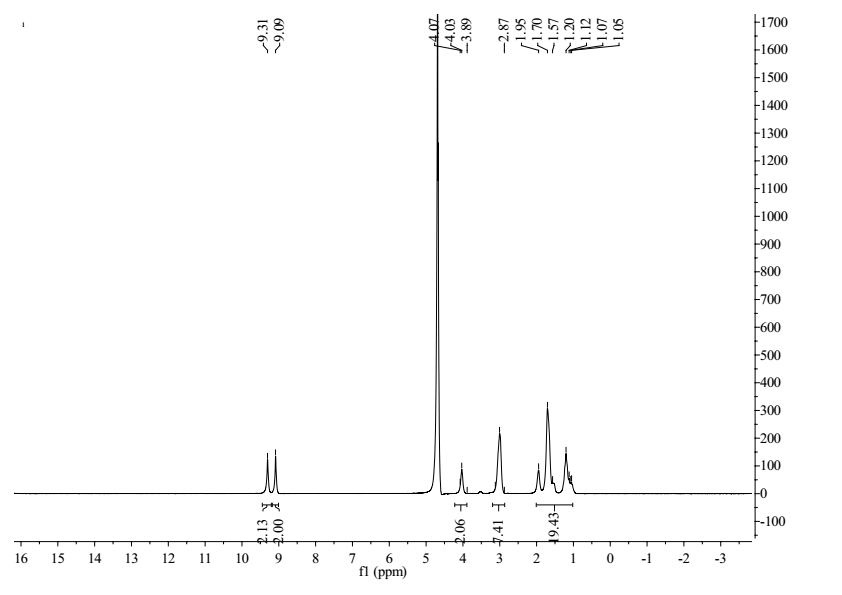


**Figure S5.** ^1^H NMR spectrum of compound **5b**.


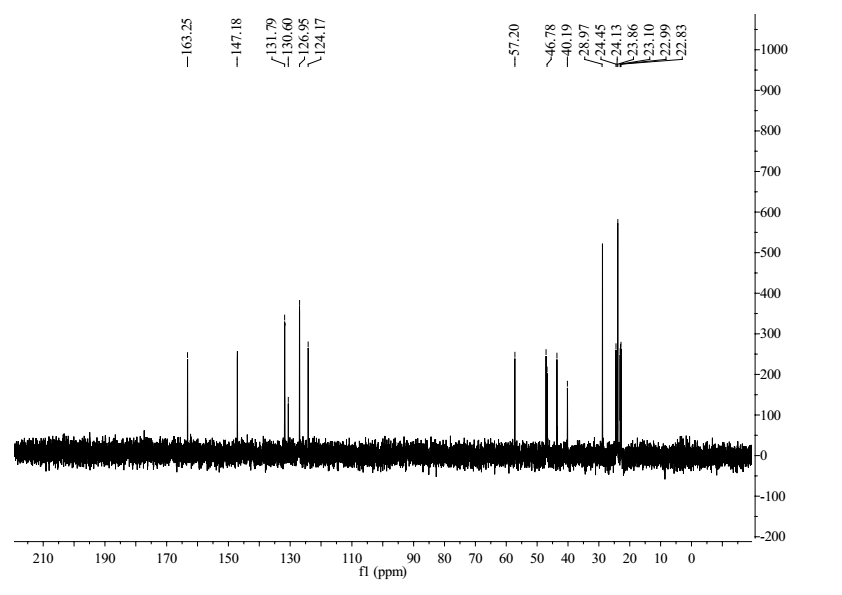


**Figure S6.** ^13^C NMR spectrum of compound **5b**.


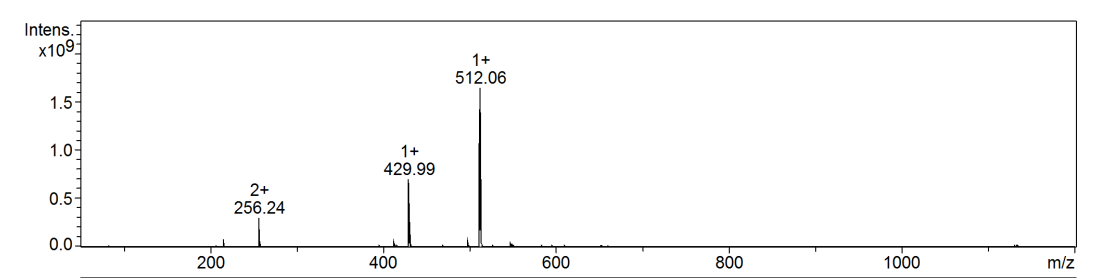


**Figure S7.** ESI mass spectrum of compound **5b**.


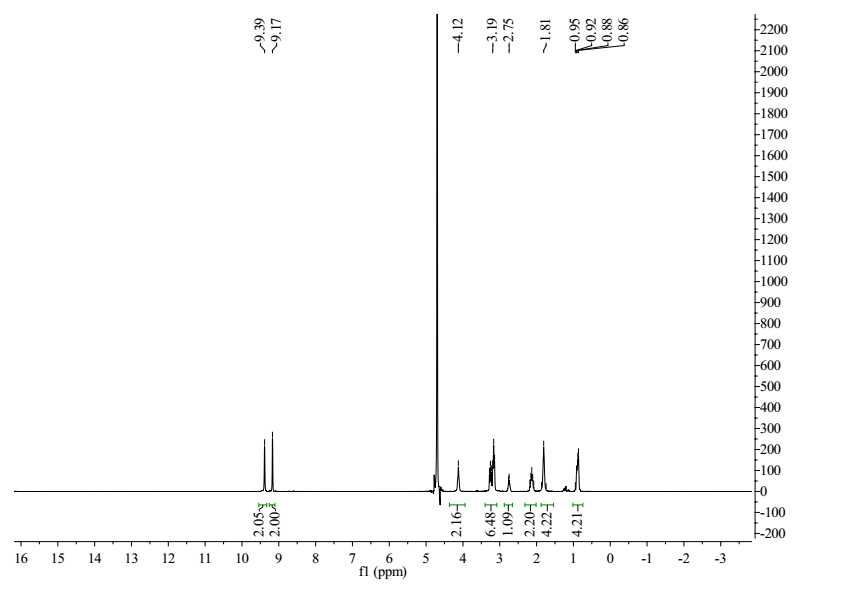


**Figure S8.** ^1^H NMR spectrum of compound **5c**.


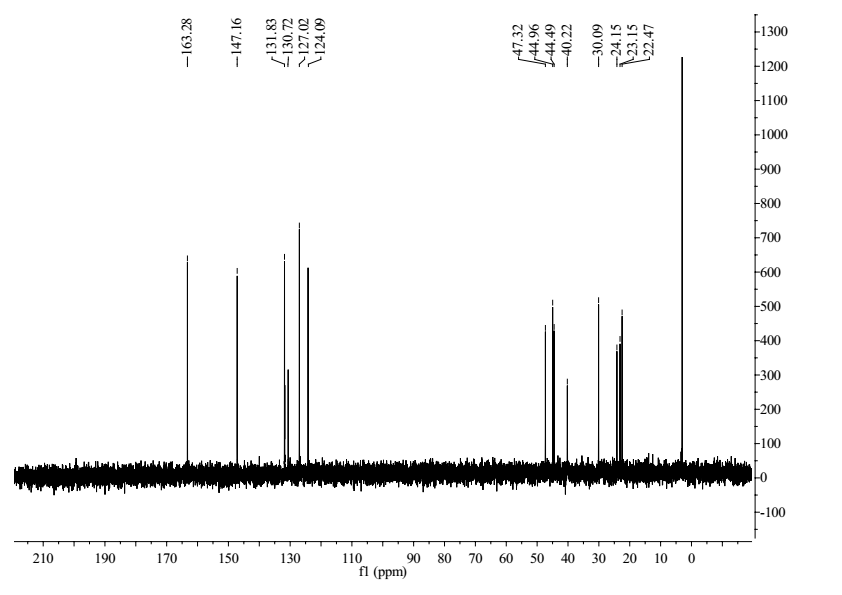


**Figure S9.** ^13^C NMR spectrum of compound **5c**.


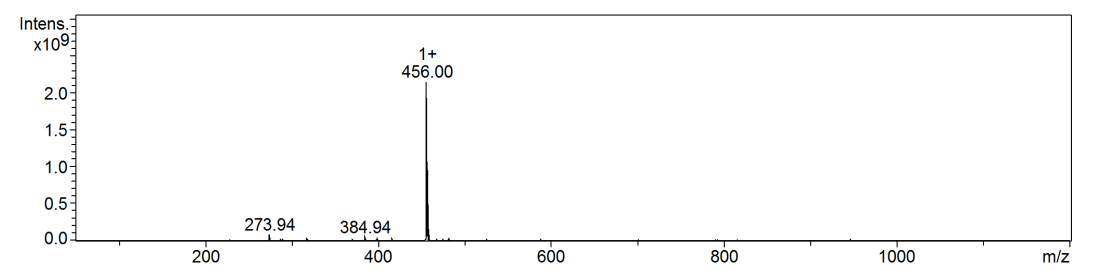


**Figure S10.** ESI mass spectrum of compound **5c**.


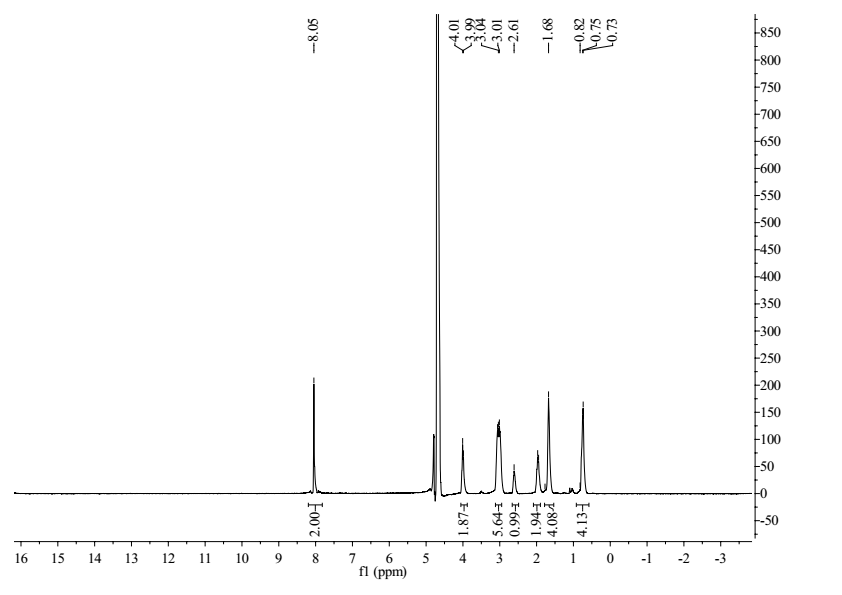


**Figure S11.** ^1^H NMR spectrum of compound **7a**.


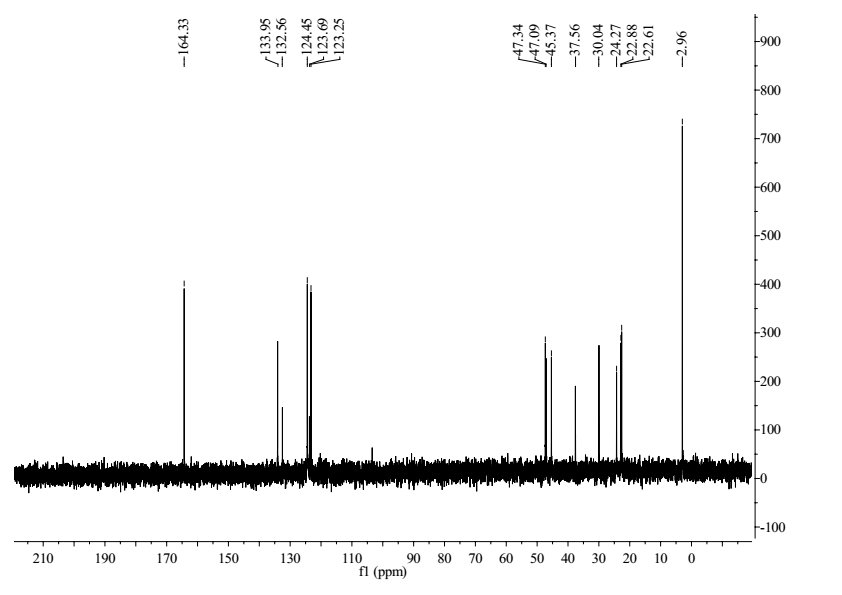


**Figure S12.** ^13^C NMR spectrum of compound **7a**.


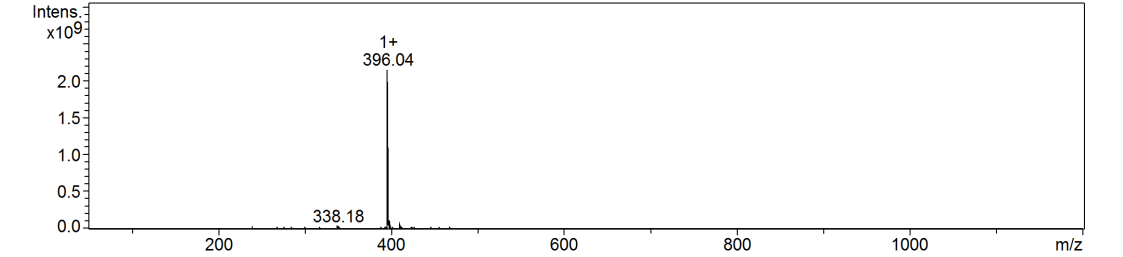


**Figure S13.** ESI mass spectrum of compound **7a**.


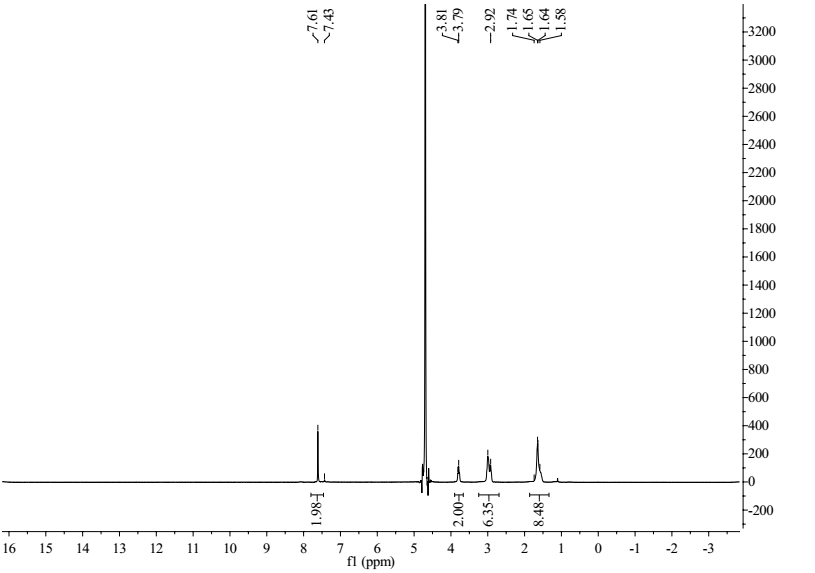


**Figure S14.** ^1^H NMR spectrum of compound **7b**.


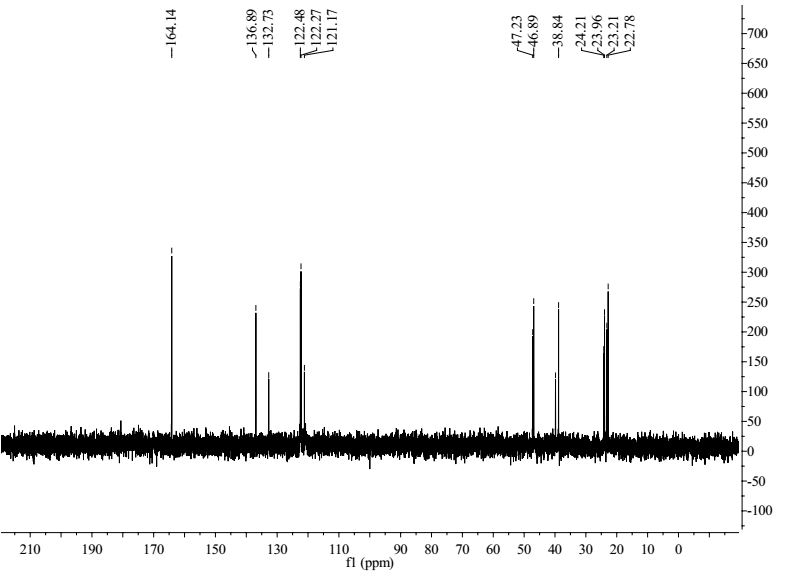


**Figure S15.** ^13^C NMR spectrum of compound **7b**.


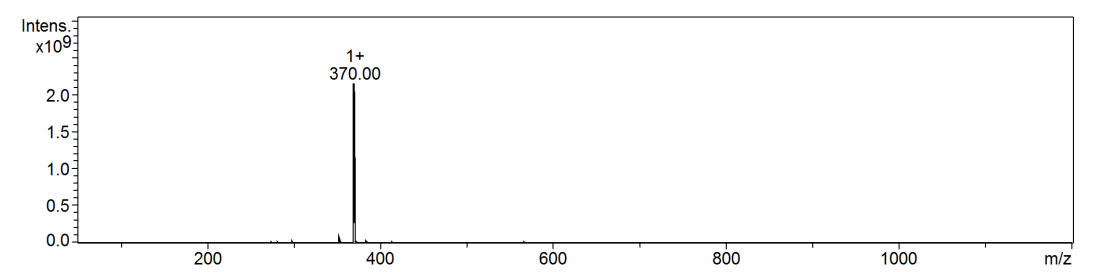


**Figure S16.** ESI mass spectrum of compound **7b**.


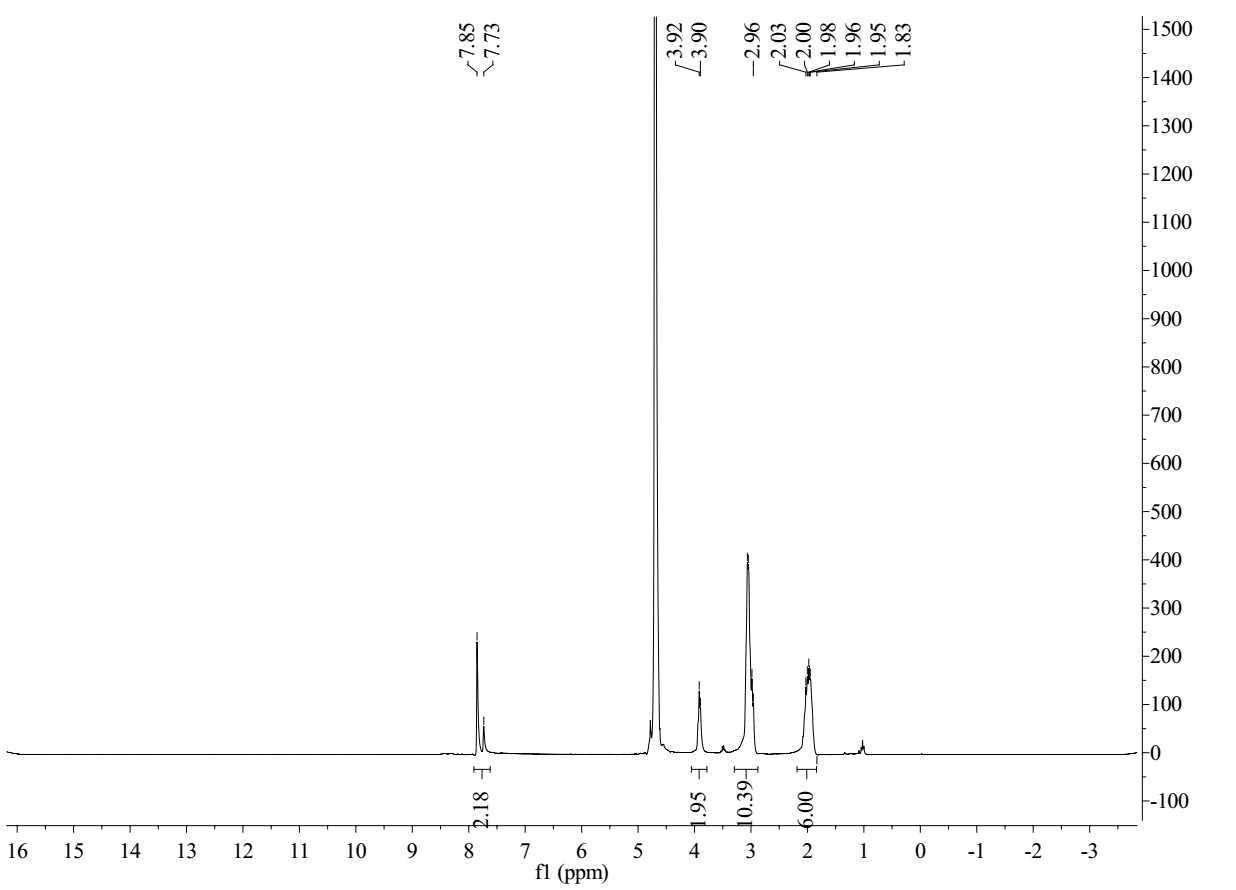


**Figure S17.** ^1^H NMR spectrum of compound **11a**.


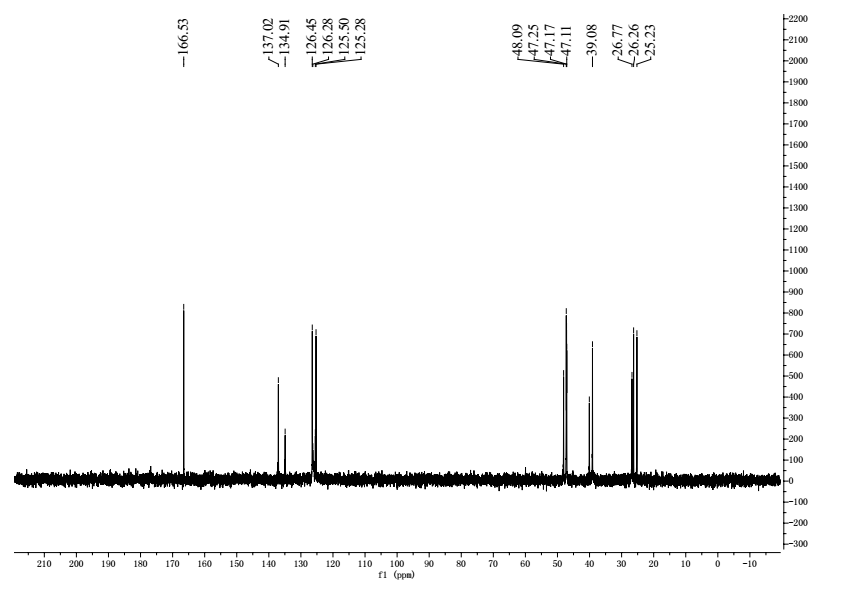


**Figure S18.** ^13^C NMR spectrum of compound **11a**.


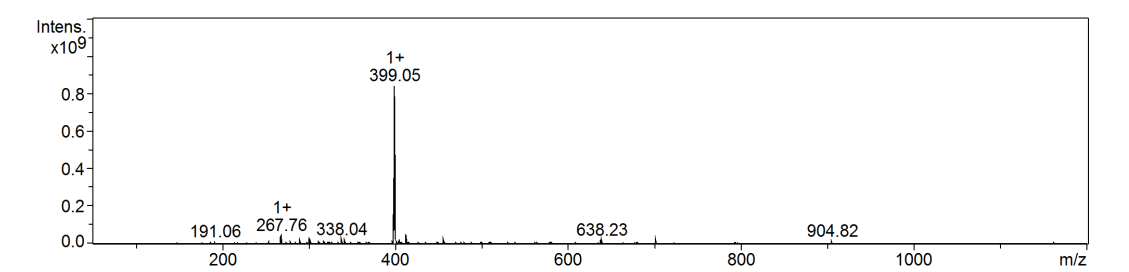


**Figure S19.** ESI mass spectrum of compound **11a**.


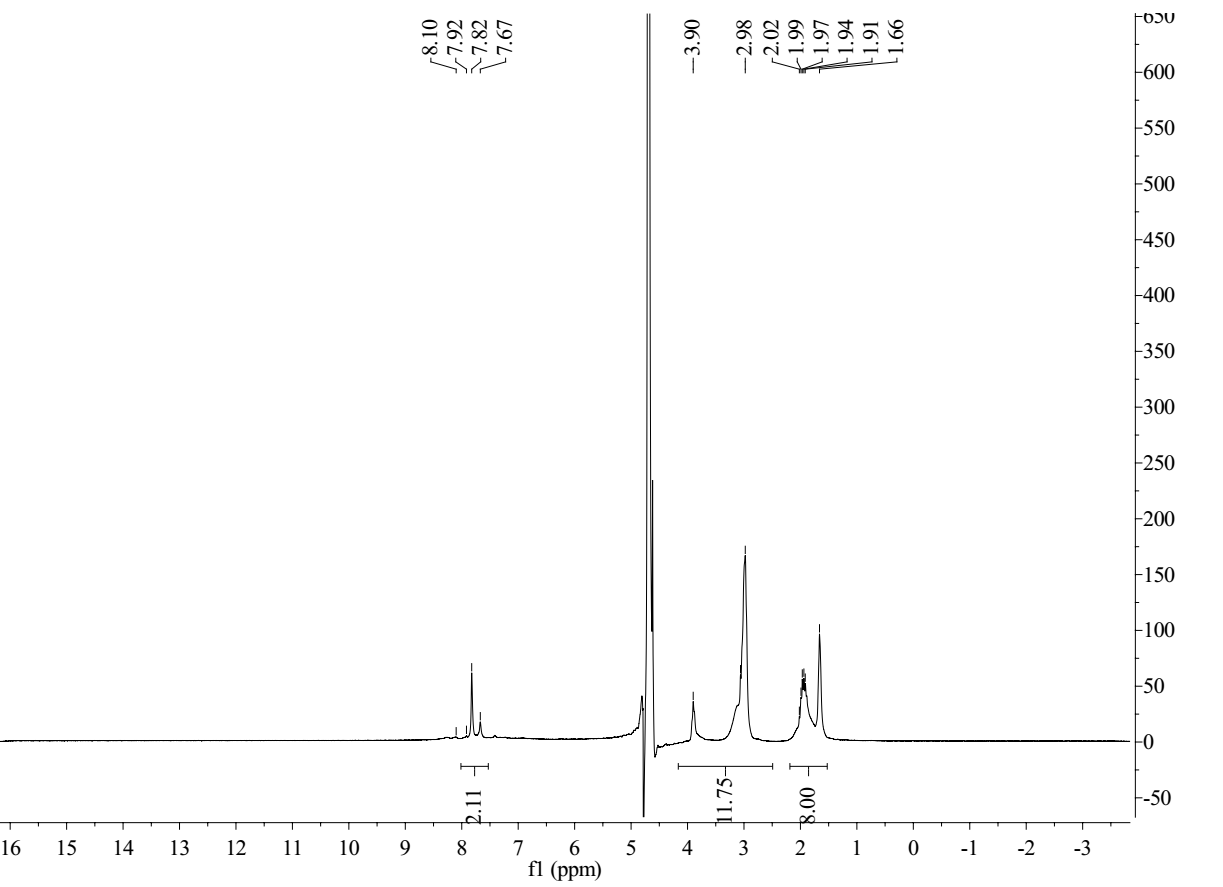


**Figure S20.** ^1^H NMR spectrum of compound **11b**.


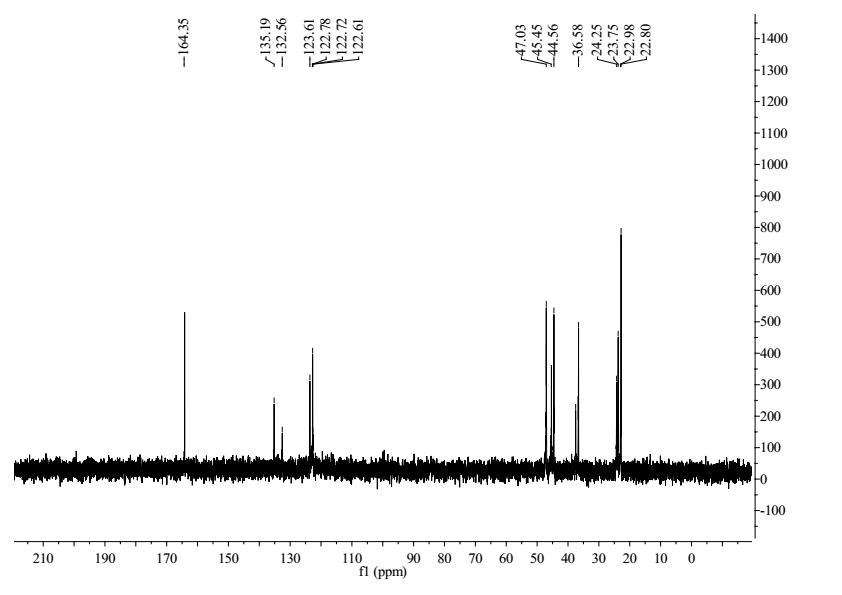


**Figure S21.** ^13^C NMR spectrum of compound **11b**.


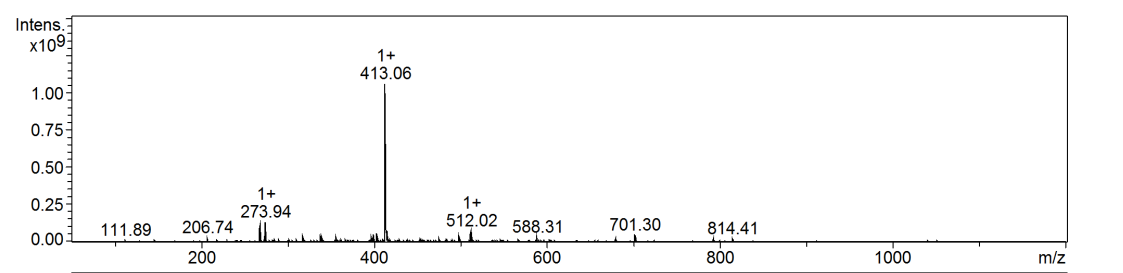


**Figure S22.** ESI mass spectrum of compound **11b**.


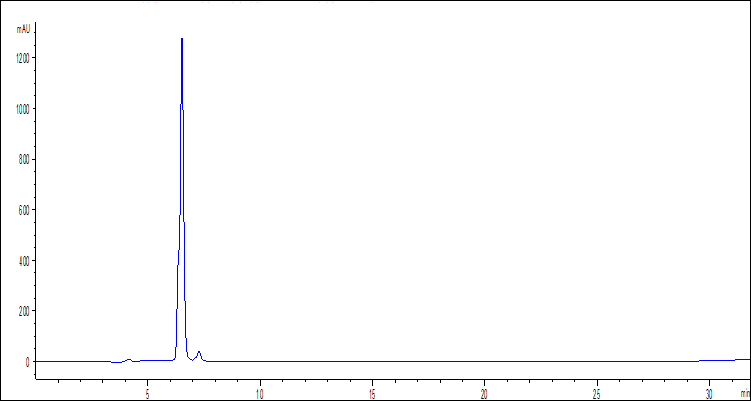


C.

**Figure S23.** Stability of compounds **5c** in water tested by RP-HPLC at room temperature. A). **5c** in water after 24h. C). Stability of compounds **5c** in water.
